# MAN1B1-CDG: Three new individuals and associated biochemical profiles

**DOI:** 10.1016/j.ymgmr.2021.100775

**Published:** 2021-06-02

**Authors:** Soraya Sakhi, Sophie Cholet, Samer Wehbi, Bertrand Isidor, Benjamin Cogne, Sandrine Vuillaumier-Barrot, Thierry Dupré, Trost Detleft, Emmanuelle Schmitt, Bruno Leheup, Céline Bonnet, François Feillet, Christine Muti, François Fenaille, Arnaud Bruneel

**Affiliations:** a AP-HP, Biochimie Métabolique et Cellulaire, Hôpital Bichat-Claude Bernard, Paris, France; b Université Paris-Saclay, CEA, INRAE, Département Médicaments et Technologies pour la Santé (DMTS), MetaboHUB, F-91191 Gif sur Yvette, France; c Service de Pédiatrie, Centre hospitalier de Versailles, Le Chesnay, France; d Centre Hospitalier Universitaire de Nantes, Service de Génétique Médicale, 44093 Nantes, France; e Université de Nantes, CNRS, INSERM, l'institut du thorax, 44000 Nantes, France; f Laboratoire CERBA, 95310 Saint-Ouen l'Aumone, France; g Service de Neuroradiologie Diagnostique et Thérapeutique, Centre Hospitalier Universitaire de Nancy, Nancy, France; h Centre de Référence Syndromes Malformatifs et Anomalies du Développement - Service de Génétique Clinique, Centre Hospitalier Universitaire de Nancy, F-54000 Nancy, France; i Laboratoire de Génétique, Centre Hospitalier Universitaire de Nancy, F-54000 Nancy, France; j Reference Center for Inborn Errors of Metabolism, University Hospital of Nancy, F-54000 Nancy, France; k Unité de Génétique Constitutionnelle, Service de Biologie, Centre Hospitalier de Versailles, Le Chesnay, France; l INSERM UMR1193, Mécanismes cellulaires et moléculaires de l'adaptation au stress et cancérogenèse, Université Paris-Sud, Châtenay-Malabry, France

**Keywords:** CDG, Hypersialorrhea, Intellectual disability, MAN1B1, N-glycan mass spectrometry, 2-DE, two-dimensional electrophoresis, A1AT, α1-antitrypsin, ApoC-III, apolipoprotein C-III, BMI, body mass index, CDG, congenital disorder(s) of glycosylation, CE, capillary electrophoresis, DD, developmental delay, DWI, Diffusion-weighted imaging, Endo H, *endo*-ß-*N*-acetylglucosaminidase H, ER, endoplasmic reticulum, ESI-QTOF, electrospray ionization – quadrupole time of flight, FLAIR, fluid-attenuated inversion recovery, HPLC, high performance liquid chromatography, Hpt, haptoglobin, ID, intellectual disability, M6, Man_6_GlcNAc_2_, M8A/B/C, Man_8_GlcNAc_2_ lacking the first/middle/third terminal mannose, M9, Man_9_GlcNAc_2_, MALDI-TOF, matrix assisted laser desorption/ionization – time of flight, Man, mannose, MRI, magnetic resonance imaging, MS, mass spectrometry, PNGase F, peptide-N-glycosidase F, Trf, transferrin, WES, whole exome sequencing

## Abstract

Congenital disorders of glycosylation (CDG) constitute an ever-growing group of genetic diseases affecting the glycosylation of proteins. CDG individuals usually present with severe multisystem disorders. MAN1B1-CDG is a CDG with nonspecific clinical symptoms such as intellectual deficiency and developmental delay. Although up to 40 affected individuals were described so far, its final diagnosis is not straightforward using common biochemical methods due to the trace-level accumulation of defective glycan structures. In this study, we present three unreported MAN1B1-CDG individuals and propose a decision tree to reach diagnosis using a panel of techniques ranging from exome sequencing to gel electrophoresis and mass spectrometry. The occurrence of MAN1B1-CDG in patients showing unexplained intellectual disability and development delay, as well as a particular transferrin glycosylation profile, can be ascertained notably using matrix assisted laser desorption/ionization – time of flight (MALDI-TOF) mass spectrometry analysis of *endo*-β-acetylglucosaminidase H-released serum N-glycans. In addition to reporting new pathogenic variants and additional clinical signs such as hypersialorrhea, we highlight particular biochemical features of MAN1B1-CDG with potential glycoprotein-specific glycosylation defects.

## 1 Introduction

**Fig. 1:**
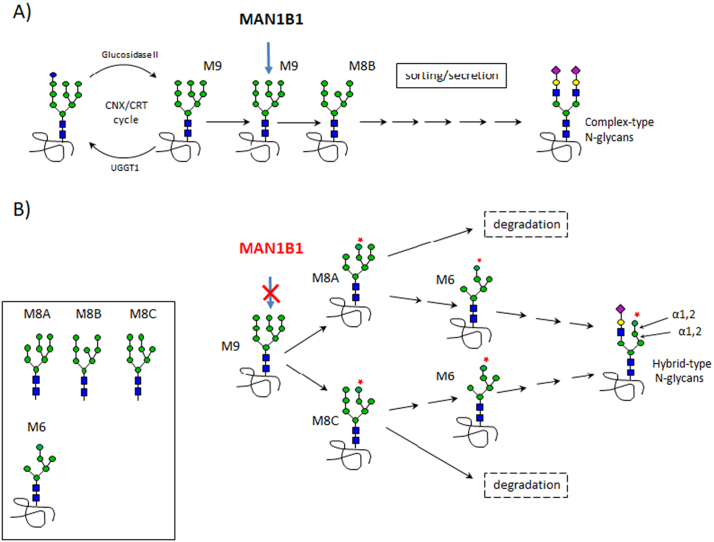
Involvement of the MAN1B1 enzyme in the secretory pathway. A) After the calnexin/calreticulin (CNX/CRT) cycle, MAN1B1 removes the Man residue of the middle branch of the GlcNAc2Man9 (“M9”) glycan linked to proteins. Glycoproteins harboring the resulting “M8B” structure are preferentially directed towards sorting and secretion. B) In case of MAN1B1 deficiency, the glycoproteins harboring the “M8A” or the “M8C” glycan structures are more likely degraded but could also result (via the schematized “M6” moiety) in glycoproteins harboring abnormal hybrid-type oligosaccharides.

Congenital disorders of glycosylation (CDG) are rare genetic diseases affecting either the synthesis of N-glycan chains in the endoplasmic reticulum (CDG-I) or, once attached to a nascent protein, their processing in the endoplasmic reticulum (ER) and the Golgi apparatus (CDG-II). Among CDG-II, MAN1B1-CDG is one of the more frequent, with up to 40 described cases [1]. It corresponds to a deficiency in α1,2-mannosidase, which catalyzes the removal of the terminal mannose (Man) from the middle branch of the Man_9_GlcNAc_2_ oligosaccharide (M9) linked to a nascent protein (Fig. 1A). After the calnexin/calreticulin cycle of the protein quality control, this Man removal generates the ‘M8B’ structure, preferentially orientating glycoproteins towards sorting and secretion. In contrast, glycoproteins bearing N-glycans lacking the terminal Man from the first or the third branch (‘M8A’ or ‘M8C’, respectively) are more likely degraded (Fig. 1B) [2].

Clinically, described MAN1B1-CDG individuals show intellectual disability (ID), developmental delay (DD), and facial dysmorphic features along with more or less frequent symptoms including hypotonia, truncal obesity and abnormal brain MRI [1]. Given these unspecific clinical signs, there is a need for efficient biological screening tests easily moving towards MAN1B1-CDG diagnosis. CDG-II are commonly detected using isoelectric focusing or capillary electrophoresis (CE) or HPLC analysis of transferrin (Trf) showing decreased level of the tetrasialo glycoform (4-sialo Trf) classically accompanied by the increases of tri-, di-, mono- and a-sialo Trf glycoforms [3]. CDG-II N-glycan abnormalities could be deeper characterized by mass spectrometry (MS) techniques. For instance, both rather high-throughput analysis of immunopurified intact Trf by electrospray ionization quadrupole time of flight mass spectrometry (ESI-QTOF) [4], as well as the analysis of enzymatically cleaved serum N-glycans by MALDI-TOF MS could precisely highlight CDG-II-related N-glycans structural defects [3]. Indeed, it could either show modified relative abundances of some specific N-glycan structures highlighting a specific enzymatic deficiency (as in MGAT2-CDG) or accumulation of multiple abnormal or truncated N-glycan structures often corresponding to an overall Golgi homeostasis dysfunction, as in ATP6V0A2-CDG (Golgi acidification defects) [5] or COG-CDG (trafficking defects) [6]. In the latter CDG, Golgi homeostasis disturbances also alter, beside N-glycosylation, the O-glycosylation pathway as demonstrated by analysis of apolipoprotein C-III (apoC-III) glycosylation [7]. In MAN1B1-CDG, Trf glycosylation patterns systematically showed a decrease of the 4-sialo Trf and a marked increase of the 3-sialo Trf glycoform, without noticeable alteration of other hyposialylated Trf glycoforms levels [8]. Besides this rather confusing Trf pattern suggestive of a potential protein variant, MALDI-TOF MS profiles of total serum/plasma N-glycans following conventional peptide-N-glycosidase F (PNGase F) treatment only produce very low intensity *m/z* signals representative of some diagnostic hybrid-type N-glycan structures [8]. To overcome these drawbacks, alternative strategies (more or less accessible in clinical settings) have been proposed such as targeted analysis of immunopurified Trf and/or IgG [4,9] as well as the convenient detection of high-mannose and hybrid-type N-glycans following Endo-β-*N*-acetylglucosaminidase (Endo H) serum treatment. Indeed, Duvet et al. [10] reported unambiguous profiles of Endo H-released serum N-glycans for seven MAN1B1-CDG patients with clear accumulation of oligosaccharidic structures harboring 4–5 Man residues.

In this work, we present three unreported MAN1B1-CDG individuals with some particular clinical signs and new pathogenic variants. Furthermore, we delineate and discuss the diagnosis pipeline we have undertaken from exome sequencing (ES)-based identification of *MAN1B1* gene variants to the definitive biochemical validation, with electrophoresis-based and MALDI-TOF MS approaches, of their pathogenic causality.

## 2 Materials and methods

### 2.1 Patients

The three described individuals were addressed to specialized consultation in the main clinical context of unexplained developmental delay (DD)/intellectual disability (ID). Informed consents for participation and sample collection were obtained via protocols approved by the ethics board committee of each concerned hospital.

### 2.2 Serum samples

Blood samples were collected in tubes without any anticoagulant to allow clot formation and sera were obtained after centrifugation for 15 min at 2000*g*.

### 2.3 Molecular analysis

Venous blood was obtained for DNA extraction from affected individuals.

For patient 1, whole exome sequencing (WES) was performed by a certified lab (CERBA Laboratory, France) using the SureSelectXT Clinical Research Exome method (Agilent) with the generation of 75-bp paired-end reads on the NextSeq500 platform (Illumina). Sequencing data were analyzed in an informatic pipeline according to Thevenon et al. [11]. The homozygous gene variant was predicted to be deleterious by dedicated *in-silico* softwares (i.e., DANN, GERP, Mutation Assessor, Mutation taster, Provean). It was confirmed by Sanger sequencing in the affected individual and was retrieved at the heterozygous state in his mother.

For patient 2, we performed exome sequencing on the proband with the SeqCap EZ MedExome (Roche) according to manufacturer's instructions and we generated 75-bp paired-end reads on an Illumina NextSeq500. Fastq files were aligned to human genome hg19 with bwa mem (v0.7.3). We then called SNVs and INDELs following GATKs best practices (v3.4). We achieved an average mean target coverage of 152×. Variants were annotated using ANNOVAR and filtered with in-house scripts to keep variants with at least 9 reads and with a variant read frequency over 20% impacting exonic sequences or splice sites (+/− 10 bp from the junction) and with an allele frequency <0.5% in 1000 genomes, genome aggregation database (gnomAD, 123,136 exomes and 15,496 whole genome sequences; accessed on 11/10/2018) and in a local database. The possible functional impact of amino-acid changes was predicted by SIFT, PolyPhen-2 hvar and CADD score. The Alamut software (Interactive biosoftware) was used to study retained variant sites. Variants were confirmed by Sanger sequencing in the proband and its parents.

For patient 3, next generation sequencing (NGS; NextSeq 550, Illumina) of a panel of 397 ‘intellectual disability’ genes (list on demand) was performed. The identified MAN1B1 homozygous gene variant was confirmed by Sanger sequencing in the affected individual and was retrieved at the heterozygous state in her mother.

### 2.4 Capillary electrophoresis of serum transferrin and two-dimensional electrophoresis of glycoproteins

Separation and detection of serum Trf glycoforms were carried out as previously described [12] using the CE Capillarys CDT method (Sebia, France). In case of Trf polymorphism suspicion, 150 μL of 1:2 diluted serum (in NaCl 0.9%) was treated overnight by 0.16 U of sialidase (Sigma) in 5 μL of 50 mM sodium phosphate buffer (pH 6.0) and then re-analyzed. Two-dimensional electrophoresis (2-DE) of Trf, haptoglobin (Hpt), α1-antitrypsin (A1AT) and apoC-III was conducted as previously described [13].

### 2.5 Mass spectrometry-based profiling of serum N-glycans

Sample preparation for N-glycomic profiling of serum samples was essentially carried out as described before [4,13]. Serum samples (5 μL) were diluted in sodium phosphate buffer (pH 7.4) and dithiothreitol solutions (20 mM and 10 mM final concentrations, respectively), and then heated at 95 °C for 5 min. After cooling, 2 μL of a 1 U/μL Peptide N-glycosidase F solution (PNGase F, Roche Diagnostics) were added and the digestion allowed to proceed overnight at 37 °C. For Endo H treatment, 5 μL of serum samples were diluted in sodium acetate (pH 4.5) and dithiothreitol (10 mM final concentrations) and heated for 5 min at 95 °C. Mixtures were then allowed to cool down at room temperature before the addition of 2 μL of a 5 mU/μL Endo H solution (Sigma-Aldrich) and a further overnight incubation at 37 °C.

After sample acidification with 5 μL of a 1 M HCl solution, proteins were precipitated in both types of digests using 150 μL of ice-cold ethanol, and incubation for 1 h. at −20 °C. Released N-glycans were then purified using porous graphitic carbon solid phase extraction cartridges (Thermo Scientific, les Ulis, France), and subsequently permethylated, purified on C18 spin-columns (Thermo Scientific), dried down and resuspended in 10 μL of a 50% methanol solution. For analysis by Matrix-assisted laser desorption/ionization time-of-flight mass spectrometry (MALDI-TOF MS), 0.5 μL of the sample was then spotted on the MALDI target and thoroughly mixed on-target with 0.5 μL of a 2,5-dihydroxybenzoic acid solution (10 mg/mL in 50% methanol containing 10 mM sodium acetate). Mass spectra were acquired using an UltrafleXtreme instrument (Bruker Daltonics) operating in the positive reflector ion mode. The spectra were obtained by accumulating 1000–5000 shots (depending on the samples) over the 500–5000 *m/z* range. Manual assignment of N-glycans was done from MS and MS/MS data on the basis of previously identified structures [14] and using the GlycoWorkBench software [15].

## 3 Results

### 3.1 Clinical reports

*Patient 1* (P1) is a 6 years old boy (Fig. S1); he is the second child of consanguineous parents of North African origin. His older sibling and parents are healthy. P1 was conceived through artificial insemination and was born following a normal at term pregnancy with a weight of 3.6 kg. At a few months of age, he presented with hypotonia and DD. He sat at 12 months and started walking at 24 months. At 1.5 years old, head circumference was 49.5 cm (+ 1 SD). He showed speech delay and was able to pronounce isolated words at 26 months. Anxiety, misophonia episodes and intermittent hypersialorrhea were noticed. At 4.5 years old, physical examination showed epicanthus, hypertelorism, plagiocephaly, joint hyperlaxity, overlapping toes and severe truncal obesity; he weighed 27.8 kg (+ 9.19 SD), and measured 119.3 cm (+ 3.17 SD). Biochemical tests at 1 year of age only showed an increase of ASAT (73 U/L; ALAT 13 U/L; *N* < 40 U/L) and blood count was normal. Brain MRI was normal. P1 is now enrolled in a mainstream school, with special education.

*Patient 2* (P2) is a 5 years old girl born as the first child of healthy non-consanguineous parents of Caucasian origin. She was born following a normal at term pregnancy, with a weight of 3.4 kg. At 12 months, she presented with a delayed motor development and hypotonia. She sat at 14 months and started walking at 28 months. She also showed speech delay and she is now able to use a few words, but mainly communicates using non-verbal language. Noticeably, P2 showed important drooling due to oral-facial hypotonia and needing subcutaneous scopolamine injections. She showed mild dysmorphic facial features, with enophthalmos and large low set ears, but also posterior plagiocephaly, joint hyperlaxity and *pectus excavatum.* Fingers are long and thin. At 3.5 years, she weighed 14 kg (− 0.47 SD), heighted 97 cm (+ 0.2 SD) with a head circumference of 50 cm. (+ 0.5 SD). ASAT were high (86 U/L) with normal ALAT (26 U/L); brain MRI was normal. There is no behavioral concern, but she requires the help of a classroom assistant.

*Patient 3* (P3) is a 13 years old female individual. She is the first child of two consanguineous parents (first cousins) of Moroccan origin. She was born at term after a normal pregnancy; she weighed 3.25 kg and heighted 51 cm. She walked at 18 months. She showed an important speech delay with first words at 2 years of age. At 9 years of age, her weight was 33 kg (+2.65 SD), her height 124.4 cm (− 1.28 SD), BMI: 21.3 kg/m^2^ (+ 3.3 SD) and head circumference 54 cm (+ 1.38 SD). No morphological particularities were reported. At that time, she attended a medico educational institute, without reading and with only a few letters recognized.

At 13 years of age, her height was 145 cm (− 1.86 SD); weight, 59 kg, BMI, 28.06 kg/m^2^ (+ 4.45 SD) corresponding to progressive truncal obesity (Fig. S2). Intellectual disability, global DD and speech delay were confirmed*.* Mild facial particularities were noted, including thin lateral eyebrows and thin upper lip. Transaminase levels were normal.

Two brain MRI performed at 9 and 13 years of age both showed a slight stable hypersignal on T2-FLAIR weighted imaging of the supratentorial white matter, mainly in the posterior areas. On the first MRI, diffusion weighted imaging (DWI) showed an unspecific thin linear hyperintensity in contact with posterior and occipital ventricular horns (Fig. S3).

### 3.2 Molecular analysis

ES was performed in P1 and P2. NGS of a panel of 397 genes (ID panel) was performed in P3. They allowed the identification of four probably pathogenic *MAN1B1* variants. In P1, a homozygous missense c.1210G>A:p.(Glu404Lys) variant was found. In P2, compound heterozygous *MAN1B1* variants were identified: a missense variant c.1581C>G:p.(Cys527Trp) inherited from the father and a stop variant c.244C>T:p.(Gln82*) from the mother. In P3, a homozygous missense variant was identified: c.1009G>A:p.(Gly337Arg). All variants were absent from the gnomAD database and have been classified as ‘likely pathogenic’ according to ACMG standards and *in-silico* prediction softwares. Furthermore, they have not been reported before.

### 3.3 Biochemical studies of serum glycoproteins

**Fig. 2:**
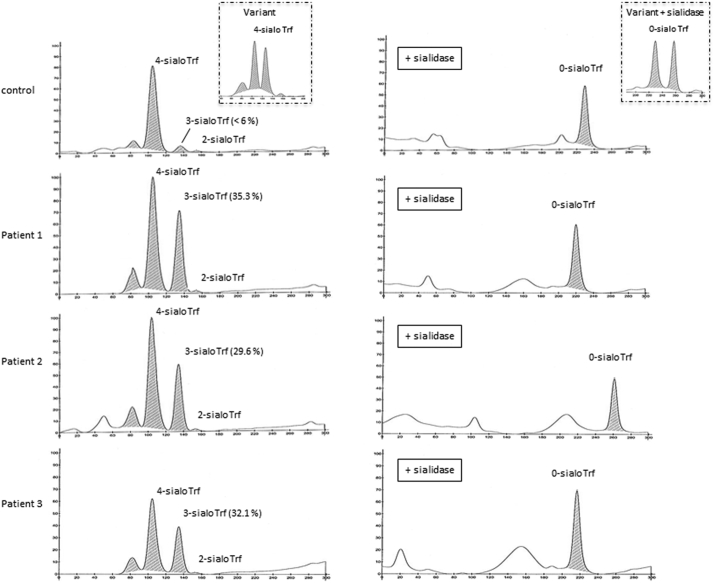
Capillary electrophoresis (CE) transferrin patterns of control and MAN1B1-CDG patients' sera. A) Compared to the control, CE transferrin (Trf) profiles of the three patients (P1, P2, P3) shared an important and isolated increase of the 3-sialo Trf fraction. These profiles are suggestive of a Trf protein variant (left rectangle). B) After neuraminidase treatment, CE Trf profiles of the patients showed one 0-sialo Trf peak, excluding a protein variant (showing two 0-sialo Trf peaks after neuraminidase treatment, as illustrated in the right rectangle). The observed shift of the asialo Trf peak of patient 2 probably results from a homozygous Trf protein variant and/or a misinterpretation of the Phoresys software.

Capillary electrophoresis (CE) of serum Trf is a well-established method for CDG screening. It essentially separates Trf glycoforms according to their terminal sialic acid (SA) content. As shown in Fig. 2A, serum Trf profiles of the three patients shared isolated and markedly abnormal increases of the 3-sialoTrf fraction (P1: 35.3%; P2: 29.6%; P3: 32.1%; normal values <6%) which might reflect at first potential Trf protein polymorphisms. The sera were then treated by sialidase to remove terminal SA and generate only 0-sialo Trf. As shown in Fig. 2B, the Trf profiles of the sialidase-treated patients' samples both showed a single peak demonstrating the absence of protein variants but instead, the occurrence of particular Trf glycosylation patterns. Thus, the three patients shared unusual CDG-II Trf profiles with isolated increases of the 3-sialo Trf glycoform. Furthermore, three serum N-glycoproteins, namely Trf, haptoglobin (Hpt) and alpha1-antitrypsin (A1AT) (Fig. S4) as well as the O-glycosylated apoC-III (Fig. S5) were analyzed using 2-DE. Whereas 2-DE corroborated the increase of the 3-sialo Trf glycoform, no evident glycosylation defect could be detected for Hpt, A1AT and apoC-III for both patients compared to controls.

### 3.4 Profiling of total serum N-glycans by mass spectrometry

**Fig. 3:**
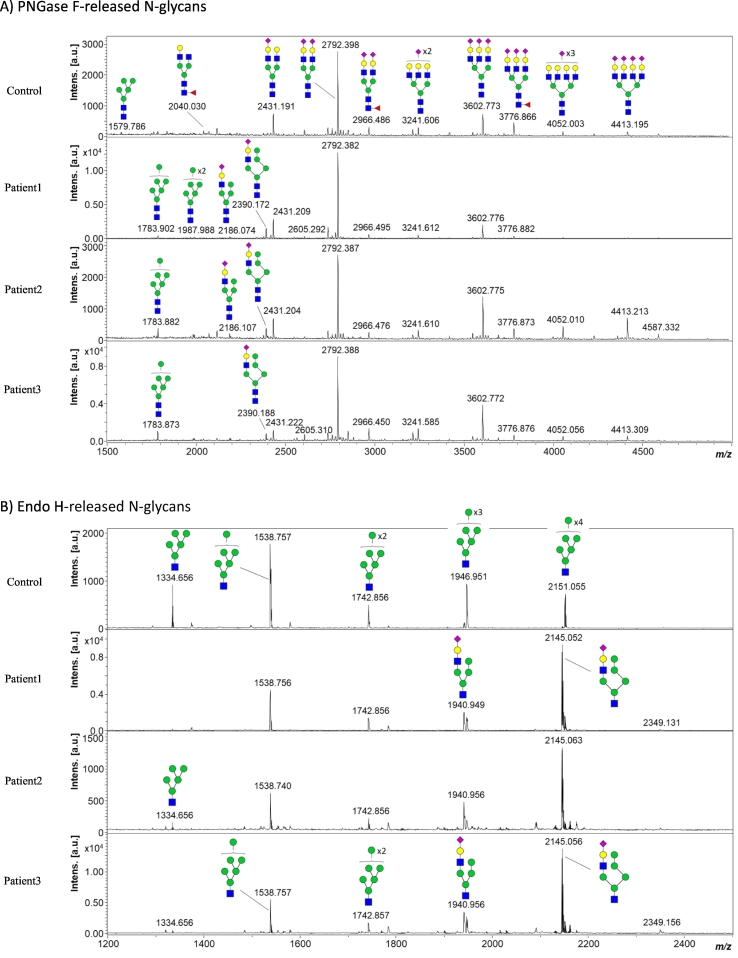
MALDI-TOF mass spectra of permethylated N-glycans released from serum samples from a healthy subject and the three MAN1B1-CDG patients following A) PNGase F, and B) Endo H treatment. Measurements were performed in the positive-ion mode and all ions are present in sodiated form. Green circles, mannose; yellow circles, galactose; blue squares, *N*-acetyl glucosamine; red triangles, fucose; purple diamonds, sialic acid. Structures of hybrid-type N-glycans at *m/z* 2390.2 (PNGase F) and *m/z* 2145.1 (Endo H) were suggested following the work of Messina et al. [17].

Total serum N-glycans were analyzed by MALDI-TOFMS following conventional PNGase F treatment (Fig. 3A). Inspection of the profiles revealed a slight but certain accumulation of some oligo- and high-mannose (at *m/z* 1783.9 and 1987.9) and hybrid-type (at *m/z* 2186.1 and 2390.2) N-glycans in the three patients when compared to a healthy subject (Fig. 3A). In good agreement with previous reports [7], the most intense hybrid-type N-glycan at *m/z* 2390.2 represents ~10% of the most abundant biantennary bi-sialylated N-glycan at *m/z* 2792.4, while altogether the high-mannose and hybrid-type species account for ~5% of the total glycan pool. Although efficient in diagnosing MAN1B1-CDG, total serum N-glycan profiling following PNGase F treatment is not optimal in terms of decision-making process due to the rather low abundance of the relevant signals. Therefore, we then evaluated the potential of the Endo H enzyme for facilitating the diagnosis of MAN1B1-CDG. Endo H specifically cleaves between the two *N*-acetyl-glucosamine residues constituting the chitobiose core of high-mannose and hybrid N-glycans. Under these conditions, five abundant mannosylated structures with Man numbers varying between 5 and 9 can be observed in the serum from the healthy subject, Man6 species at *m/z* 1538.8 being the most intense glycan structure (Fig. 3B). Such N-glycan profile appeared drastically modified for MAN1B1-CDG individuals with strong accumulation of monosialylated hybrid N-glycan species at *m/z* 1940.9 and *m/z* 2145.1 along with a pronounced relative decrease of the Man5 species (Fig. 3B).

## 4 Discussion

Clinically, like other MAN1B1-CDG individuals described so far [1], intellectual disability, development delay, speech delay and mild facial dysmorphic features (including plagiocephaly in two cases) were systematically retrieved in the three described individuals. Also in line with previous reports, truncal obesity (2/3 patients), joint hyperlaxity (2/3 patients), mild isolated increase of ASAT (2/3 patients) and abnormal brain MRI (1/3) are frequent features. Concerning truncal obesity, we showed in P3 that this symptom could be progressive pinpointing the interest of a regular BMI monitoring of MAN1B1-CDG individuals. Furthermore, we report here for the first time hypersialorrhea/excessive drooling in two patients, which expands the phenotype of MAN1B1-CDG. To the best of our knowledge and based on a recent review article about clinical findings in CDG [16], hypersialorrhea/drooling has never been reported as a dominant clinical sign in any of these inherited diseases. Thus, we think that this symptom could be relevant in the diagnosis of MAN1B1-CDG. Reassessing the symptomatology of previously reported MAN1B1-CDG individuals regarding this particular feature would be interesting.

Genetically, we expand the genotype of MAN1B1-CDG with four (three missense and one frameshift) variants not reported so far. In the context of a new pregnancy in P1 family, a prenatal diagnosis was carried out; the fetus was a homozygous carrier of the variant and a terminal medication was proposed.

Biochemically, based on the presented cases and previous reports, MAN1B1-CDG, the most common CDG-II, could be characterized by an isolated and significant increase of the 3-sialo Trf fraction, as determined using TIEF [6], CE or 2-DE (this study). Furthermore, the 2-DE analysis of two additional serum N-glycoproteins, namely Hpt and AAT did not show evident N-glycan abnormalities suggesting that only the glycosylation of a few proteins (including Trf) is significantly impacted by the MAN1B1 deficiency. In agreement with a mannosidase defect, the MALDI-TOFMS analysis of PNGase F- and Endo H-released total serum N-glycans showed that the 3-sialo Trf systematic increase is linked to elevated amounts of hybrid type N-glycans harboring 5 or 4 Man residues potentially with unusual α1,2 linkages. Although specific, those N-glycan species occur at trace levels in serum (<10% when summing all the PNGase-released species), which corroborates the intriguing involvement of a limited number of circulating N-glycoproteins. By hydrolyzing Endo H-released N-glycans with mannosidases, Duvet et al. demonstrated that MAN1B1-specific hybrid N-glycans have isomeric structures different from those present in control samples, with α1,2 linked mannose residues [10]. Recently an alternative MS-based strategy involving the analysis of Rapifluor-derivatized PNGase F-released N-glycans, Messina et al. [17] confirmed these findings in MAN1B1-CDG individuals. Thus, our data support the fact that MS-based profiling of both PNGase F- and Endo H-released serum N-glycans proved efficient in highlighting MAN1B1-CDG for individuals showing unexplained ID and isolated elevated 3-sialo Trf level. This especially holds true when considering Endo H-released N-glycans, which showed the higher sensitivity and specificity.

## 5 Conclusions

We report three new MAN1B1-CDG individuals and expand the related clinical and molecular spectrum. MAN1B1-CDG can be characterized by developmental delay/intellectual disability and an isolated increase of 3-sialo Trf linked to abnormal hybrid-type N-glycans. In case of a suggestive Trf profile, MALDI-TOF analysis of EndoH-released serum N-glycans appears as an efficient and straightforward second line-laboratory tool for facilitating the diagnosis. Further work would be required to investigate some MAN1B1-CDG characteristics associated with some apparent glycoprotein- and/or site-specific glycosylation defects.

## Appendix A Supplementary data

Supplementary material Image 1
