# Supplementary material for: MAN1B1-CDG: Three new individuals and associated biochemical profiles
Source: Mol Genet Metab Rep. 2021 Jun 2;28:100775. doi: 10.1016/j.ymgmr.2021.100775 (PMC8182421; doi:10.1016/j.ymgmr.2021.100775)
Supplement: Supplementary file 1 — Supplementary material [file mmc1.pdf]

Supplementary Figure 1

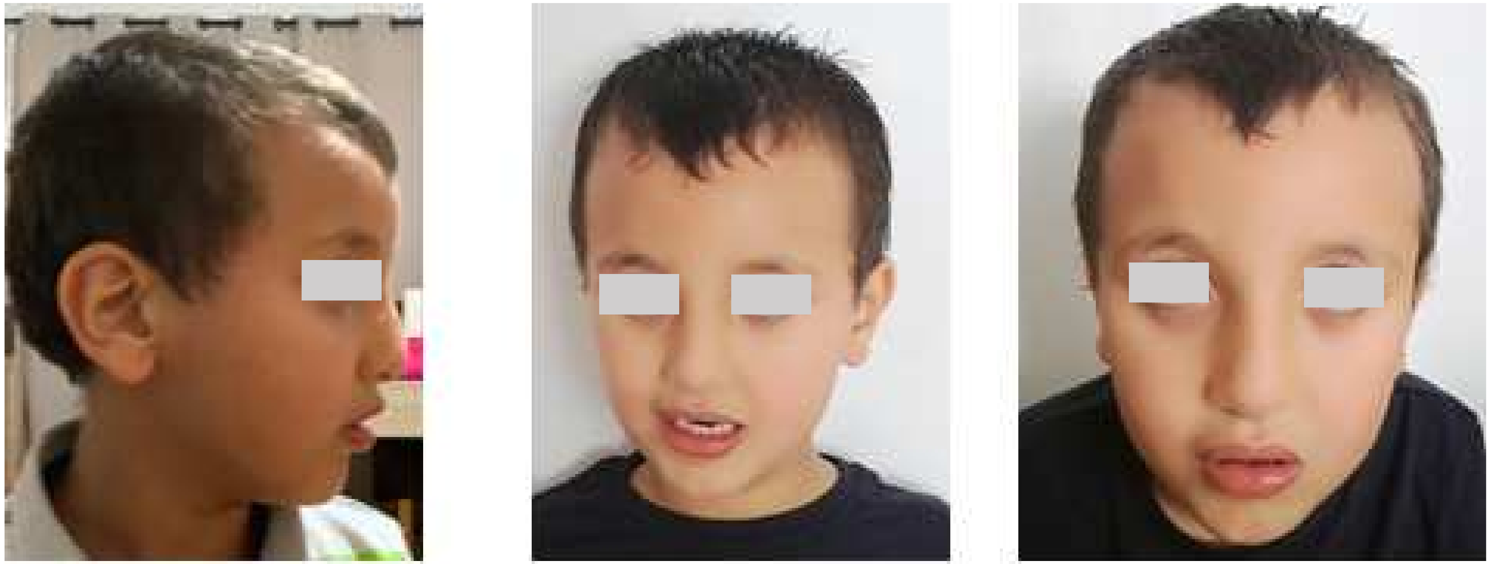

Fig. S1: Frontal and lateral photographs of individual P1 at age 5 years with facial features, macrocephaly, epicanthus, hypertelorism.

Supplementary Figure 2

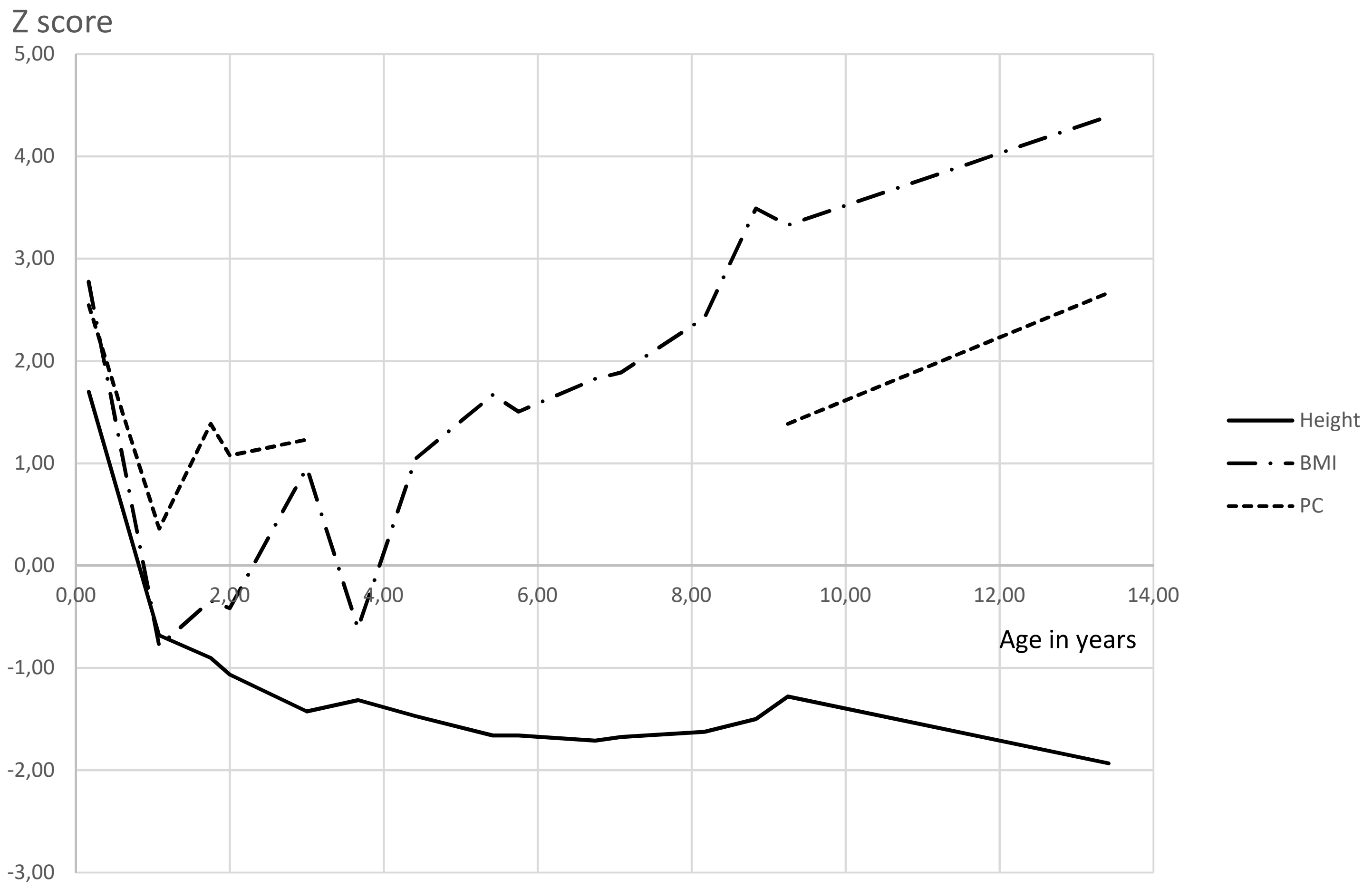

Fig. S2: Growth curves of Patient 3. With age, the body mass index (BMI) of Patient 3 progressively increased to reach abnormal level at around 7 year-old. This, by contrast with early abnormally low height.

Supplementary Figure 3

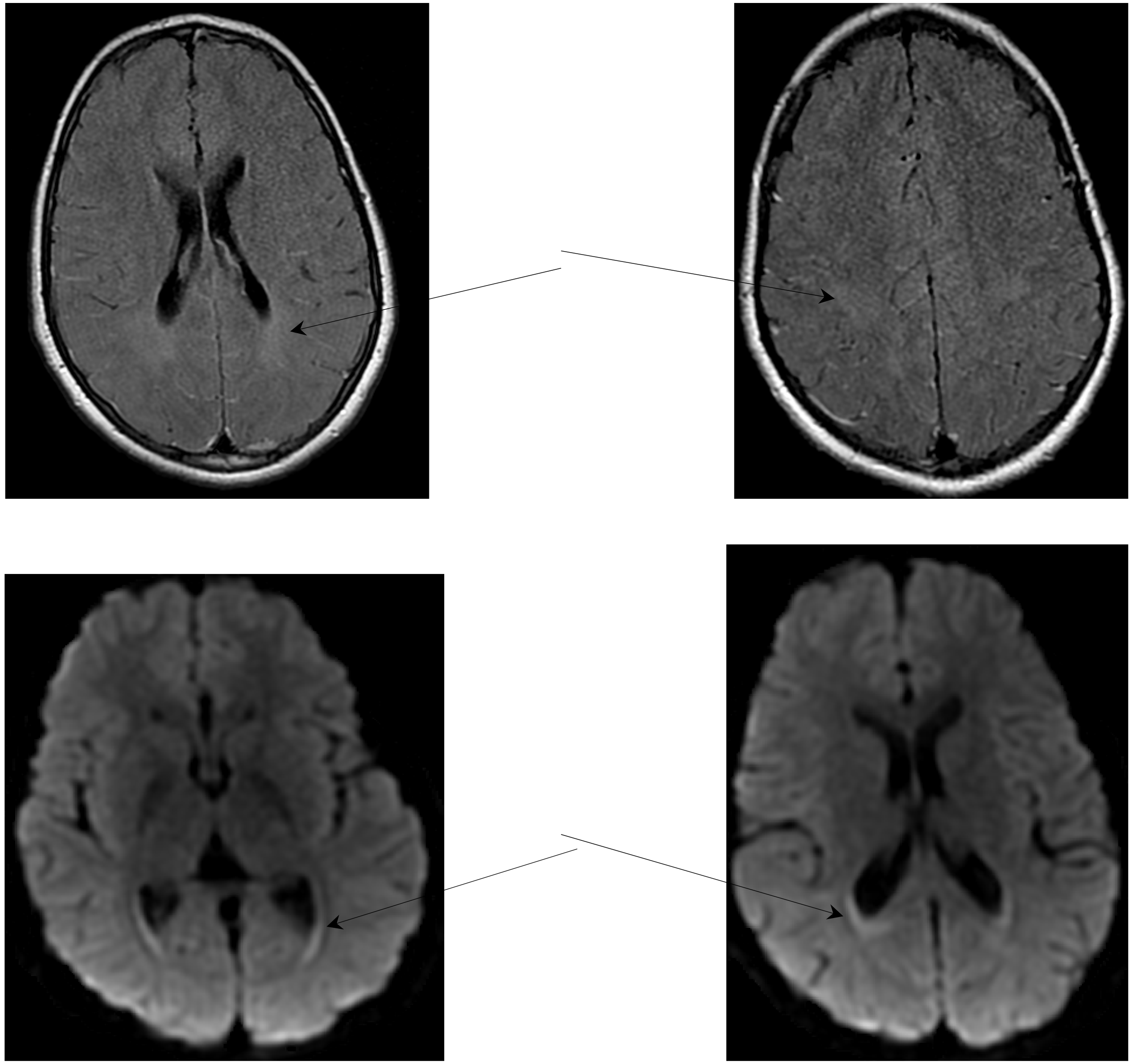

Fig. S3: Patient 3' cerebral MRI at 9 years of age. Top pictures, axial FLAIR: slight hypersignal of posterior white matter (arrows). Bottom pictures, axial DWI: thin linear hyperintensity in contact with posterior and occipital ventricular horns (arrows).

Supplementary Figure 4

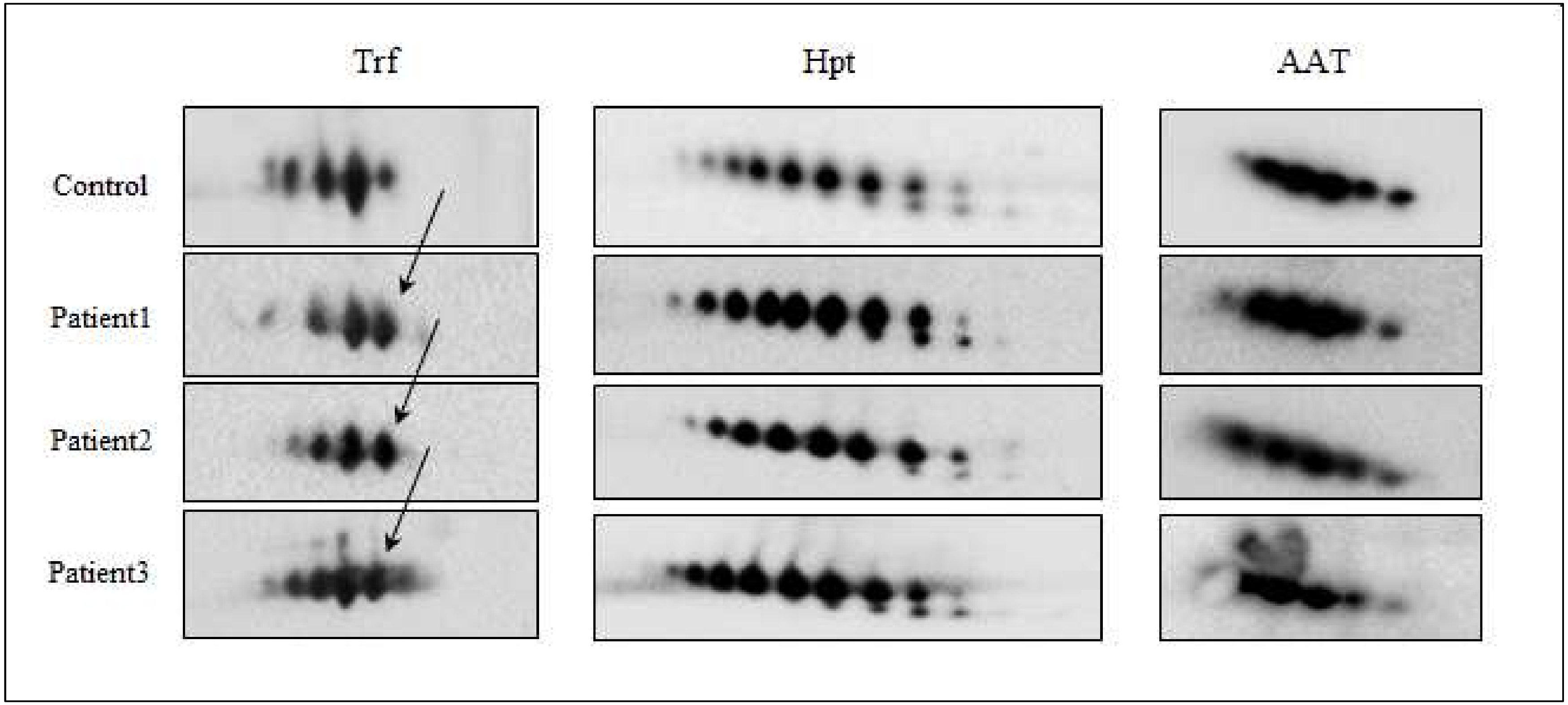

Fig. S4: 2-DE of N-glycosylated transferrin (Trf), haptoglobin (Hpt) and α1-antitrypsin (A1AT). The 2-DE patterns confirmed the isolated increase of the 3-sialo Trf fraction (black arrows) in the sera of the MAN1B1-CDG patients compared to control. Hpt and AAT patterns of the three individuals were rather indistinguishable from the control.

Supplementary Figure 5

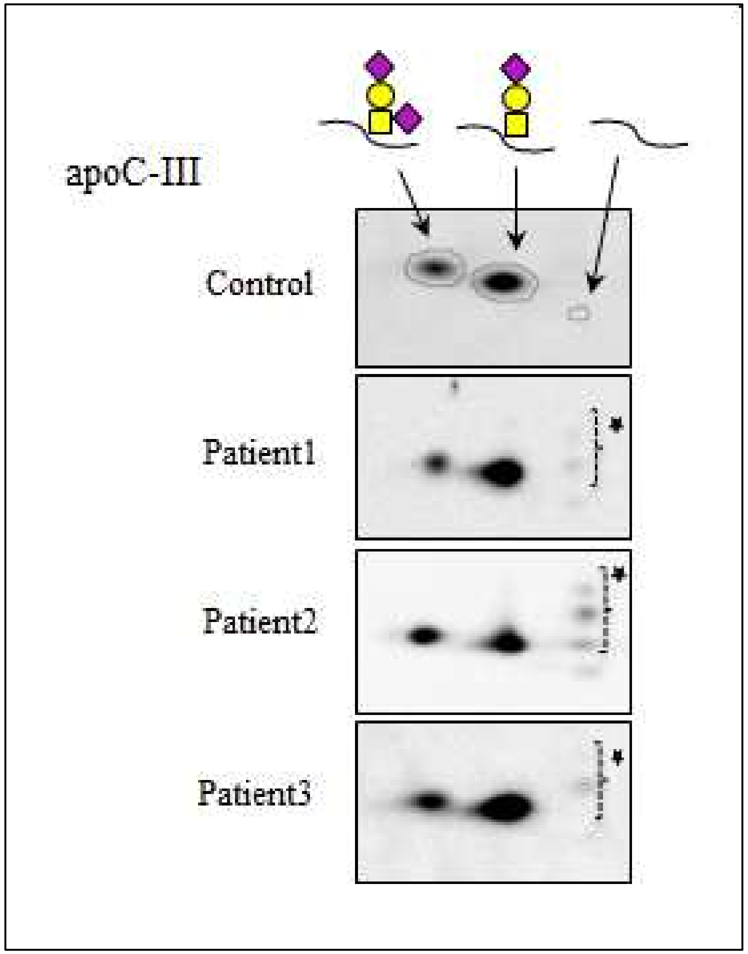

Fig. S5: 2-DE of mucin core1 O-glycosylated apoC-III. 2-DE patterns of apoC-III from the sera of the MAN1B1-CDG individuals did not show notable differences compared to control. Black stars indicate minor mucin core2 apoC-III glycoforms, not investigated in this study.
